# SOCIAL CAPITAL AND HEALTH AT OLDER AGES IN GHANA

**DOI:** 10.1093/geroni/igz038.2737

**Published:** 2019-11-08

**Authors:** Kojo Paul Ayernor

**Affiliations:** The Graduate Center, CUNY, New York, New York, United States

## Abstract

Significant increases in life expectancy and declining fertility confirms that population ageing is fast becoming a reality in several West African nations, and the demographic transition is expected to continue well into this century. This study examines the association between social capital, self-rated health, and depression among older adults aged 50 years and over in Ghana. It draws on a small scale, yet nationally representative longitudinal data from the Global Ageing Study (SAGE-WHO, 2003-2007). Social capital is conceptualized through four dimensions: personal control, generalized trust, safety in the community and free expression. Although there were not significant findings on social capital and depression, results demonstrated significant associations between social capital and self-rated health. The relationship between social capital and self-rated health suggests the need to extend and expand upon research regarding the relationship between social capital, health, and well-being in later life in aging African communities.

